# Altered Dynamic Functional Connectivity in *de novo* Parkinson’s Disease Patients With Depression

**DOI:** 10.3389/fnagi.2021.789785

**Published:** 2022-02-14

**Authors:** Jianxia Xu, Miao Yu, Hui Wang, Yuqian Li, Lanting Li, Jingru Ren, Chenxi Pan, Weiguo Liu

**Affiliations:** ^1^Department of Neurology, The Affiliated Brain Hospital of Nanjing Medical University, Nanjing, China; ^2^Department of Neurology, Lianyungang Hospital of Traditional Chinese Medicine, Lianyungang, China; ^3^Department of Neurology, Affiliated BenQ Hospital of Nanjing Medical University, Nanjing, China

**Keywords:** Parkinson’s disease, depression, dynamic functional connectivity, dynamic graph theoretical analysis, neural network

## Abstract

**Background:**

Depression is one of the most prevalent and disturbing non-motor symptoms in Parkinson’s disease (PD), with few dynamic functional connectivity (dFC) features measured in previous studies. Our aim was to investigate the alterations of the dynamics in *de novo* patients with PD with depression (dPD).

**Methods:**

We performed dFC analysis on the data of resting-state functional MRI from 21 *de novo* dPD, 34 *de novo* patients with PD without depression (ndPD), and 43 healthy controls (HCs). Group independent component analysis, a sliding window approach, followed by k-means clustering were conducted to assess functional connectivity states (which represented highly structured connectivity patterns reoccurring over time) and temporal properties for comparison between groups. We further performed dynamic graph-theoretical analysis to examine the variability of topological metrics.

**Results:**

Four distinct functional connectivity states were clustered *via* dFC analysis. Compared to patients with ndPD and HCs, patients with dPD showed increased fractional time and mean dwell time in state 2, characterized by default mode network (DMN)-dominated and cognitive executive network (CEN)-disconnected patterns. Besides, compared to HCs, patients with dPD and patients with ndPD both showed weaker dynamic connectivity within the sensorimotor network (SMN) in state 4, a regionally densely connected state. We additionally observed that patients with dPD presented less variability in the local efficiency of the network.

**Conclusions:**

Our study demonstrated that altered network connection over time, mainly involving the DMN and CEN, with abnormal dynamic graph properties, may contribute to the presence of depression in patients with PD.

## Introduction

Parkinson’s disease (PD) is the second most prevalent neurodegenerative disorder (Poewe et al., 2017). Nowadays, it is widely acknowledged that PD is characterized not only by motor features but also by a multitude of non-motor symptoms, such as depression, of which prevalence is approximately 35% (Reijnders et al., 2008). Depression can occur throughout the course of PD, and even precede the onset of motor symptoms in the prodromal stage (Berg et al., 2015). Depression in PD can worsen motor and cognitive functions, increase disability, thus, severely impairing the living quality of patients (Pfeiffer, 2016). A better understanding of the underlying pathophysiological mechanisms is crucial for early diagnosis and treatment.

Resting-state functional MRI (rsfMRI) is a novel, non-invasive method, widely used in investigating neuroimaging substrates of PD with depression (dPD). Previous studies employing the amplitude of low-frequency fluctuation (ALFF) and regional homogeneity (ReHo) approaches revealed that aberrant regional brain activity in several specific areas of prefrontal and cingulate cortices was associated with depressive symptoms in PD (Wen et al., 2013; Luo et al., 2014; Sheng et al., 2014). Functional connectivity (FC) studies demonstrated that abnormal FC between the prefrontal and limbic regions may lead to emotional dysregulation in PD (Luo et al., 2014; Hu et al., 2015; Huang et al., 2015). Recently, more studies focused on large-scale neural networks analysis and found that patients with dPD showed altered intra- and inter-network connectivity, with the involvement of the cognitive executive network (CEN), default mode network (DMN), and basal ganglia network (BGN) (Wei et al., 2017; Liao et al., 2020; Lin et al., 2020). However, most studies were based on the assumption that neural activity remained stationary over the entire scanning, overlooking the time-varying characteristics of functional activation (Chang and Glover, 2010).

In fact, the strength and directionality of FC can substantially change at fast time scales of seconds to minutes (Hutchison et al., 2013). Nowadays, temporal fluctuations can be captured at short time scales by dynamic FC (dFC) analysis, providing more detailed connectivity information within the brain (Calhoun et al., 2014). Emerging evidence revealed that dynamic FC analysis can add sensitivity to the exploration of neural activity (Keilholz, 2014) in conditions such as psychiatric disorders and neurodegenerative diseases (Filippi et al., 2019; Bolton et al., 2020). Importantly, multiple studies have identified the temporal fluctuation of functional connectivity in PD and showed that dynamic properties were associated with the severity of motor symptoms (Kim et al., 2017) and several non-motor symptoms, including cognitive function (Fiorenzato et al., 2019), impulse control disorders (Navalpotro-Gomez et al., 2020), and rapid eye movement sleep behavior disorder (Gan et al., 2021). Furthermore, dynamic graph-theoretical analysis is a novel approach for providing quantified measures reflective of information or community organization over time (Yu et al., 2015; Bolton et al., 2020). Higher variability of global efficiency was observed in patients with PD in a previous study (Kim et al., 2017). However, there is still a lack of studies on the alterations of the dynamics in patients with dPD.

Additionally, considering that prior studies have proven that levodopa and antidepressants could affect the dynamic functional measures (Arnone et al., 2018; Chen et al., 2021), we focused on drug-naïve patients with dPD, thereby eliminating the influence of medication. The aim of the study was to evaluate the differences in dynamics between patients with PD with and without depression. We hypothesized that (1) patients with dPD would show distinctive dFC patterns and temporal properties and that (2) altered dynamic topological metrics may account for the occurrence of depression in PD.

## Materials and Methods

### Participants

The study was approved by the Medical Ethics Committee of the Affiliated Brain Hospital of Nanjing Medical University. Written informed consent was obtained from all participants after a full explanation of the whole procedure. Patients were recruited from the Movement Disorder Clinic at the Department of Neurology of Brain Hospital Affiliated with Nanjing Medical University. PD was diagnosed by an experienced neurologist according to the UK Parkinson’s Disease Society Brain Bank diagnostic criteria (Hughes et al., 1992). Patients were excluded if they had (1) anti-Parkinsonism medications prior to enrollment; (2) history of cerebrovascular disorders, head injury, seizure, hydrocephalus, intracranial mass, previous neurological surgery, and other neurologic diseases; (3) meeting the diagnostic criteria for PD with dementia; (4) history of psychiatric diseases other than depression; (5) antidepressant treatment or other psychiatric therapy; (6) other major systemic comorbidities; (7) history of alcohol or drug dependency or abuse; and (8) excessive head motion and poor MRI image quality (see below). All the patients were followed up for at least one year after enrollment to confirm the diagnosis according to the disease evolution and response to the dopaminergic therapy. Healthy controls (HCs) were matched to patients with PD for age, gender, and education. This study finally included 55 *de novo* patients with PD and 43 HCs. All the participants completed the clinical assessments and MRI scans during the baseline visit.

### Clinical Assessments

Patients with dPD were diagnosed using the Diagnostic and Statistical Manual of Mental Disorders, Fifth Edition (DSM-V) criteria by an experienced psychiatrist. The severity of depression was quantified with the 17-item Hamilton Depression Rating Scale (HAMD-17). A HAMD-17 score higher than 14 was required for a more accurate diagnosis of dPD (Leentjens et al., 2000). Twenty-one patients with PD were diagnosed with dPD at last. In addition, motor symptoms and disease severity were evaluated by the motor section of the Unified Parkinson’s Disease Rating Scale (UPDRS-III) and Hoehn and Yahr (H&Y) staging scale. Cognitive function was assessed using the Mini-Mental State Examination (MMSE). All the assessments were conducted immediately before the MRI scan.

### MRI Acquisition

Magnetic resonance (MR) images were required using a 3T MRI scanner (Siemens, Verio, Germany). All participants laid supine with their heads fixed by foam pads with a standard birdcage head coil to minimize head movement. The participants were instructed to remain as still as possible and to close their eyes while remaining awake without thinking of anything. Axial anatomical images were acquired using a T1 fluid-attenuated inversion recovery sequence with the following parameters: repetition time (TR) = 2,530 ms; echo time (TE) = 3.34 ms; field of view (FOV) = 256 mm × 256 mm; matrix = 256 × 192; slice thickness/gap = 1.33/0.5 mm; flip angle (FA) = 7 degrees; bandwidth = 180 HZ/PX; 128 slices covered the whole brain, for image registration and functional localization. Functional images were subsequently collected in the same slice orientation with a gradient-recalled echo-planar imaging pulse sequence. A total of 240 volumes were obtained (TR = 2,000 ms; TE = 30 ms; FOV = 220 mm × 220 mm; matrix = 64 × 64; thickness/gap = 3.5/0.63 mm; FA = 90 degrees; bandwidth = 2,232 HZ/PX; slice numbers = 31).

### Resting-State Functional MRI Data Preprocessing

Preprocessing of the functional MRI (fMRI) data was carried out using data processing assistant for resting-state fMRI (DPARSF)^1^ (Chao-Gan and Yu-Feng, 2010) based on SPM12^2^. The first 10 volumes of each rest functional section were removed for signal equilibrium and participant adaptation to the scanning environment, resulting in a total of 230 volumes. The remaining volumes were then corrected for slice timing using the middle slice as a reference, realigned for head motion correction, segmented into white matter, gray matter, and cerebrospinal fluid (CSF) using tissue probability maps derived from the T1 images, normalized into the standard Montreal Neurological Institute space using diffeomorphic anatomical registration through exponentiated Lie algebra (DARTEL), resliced as a 3 mm × 3 mm × 3 mm voxel size, and spatially smoothed with a 6 mm full-width at half-maximum (FWHM) Gaussian kernel. Aiming to further minimize the potential effects of head motion, we excluded patients with a mean framewise displacement (FD) > 0.5 mm or whose head motion exceeded a maximum translation of 3 mm or rotation of 3° from the further analysis (1 patient with dPD, 3 patients with ndPD, and 2 HCs), referring to a previous study (Navalpotro-Gomez et al., 2020). There were no significant group differences in head motion between dPD (mean FD = 0.08 ± 0.04 mm), ndPD (mean FD = 0.10 ± 0.06 mm), and HCs (mean FD = 0.11 ± 0.06 mm) groups (ANOVA, *P* = 0.12). Additionally, considering the possible deleterious influence of head motion, we added a scrubbing step in the preprocessing procedures and repeated the following analyses for further verification (Power et al., 2012; Yan et al., 2016). we found nearly consistent results with regard to temporal properties (Supplementary Figure 2).

### Group Independent Component Analysis

The preprocessed resting-state data of all subjects were decomposed into functional networks by applying spatial group independent component analysis (ICA) implemented in the Group ICA of the fMRI Toolbox (GIFT v4.0b)^3^. Subject-specific data were reduced to 120 independent components (ICs) with the principal component analysis (PCA), and further decomposed into 100 ICs using the expectation-maximization algorithm at the group level as previously done (Kim et al., 2017). The Infomax ICA algorithm in ICASSO (Bell and Sejnowski, 1995) was repeated 20 times to ensure stability and validity (Himberg et al., 2004). Subject-specific spatial maps and time courses were obtained using the GICA back-reconstruction approach (Calhoun et al., 2001).

**FIGURE 1:**
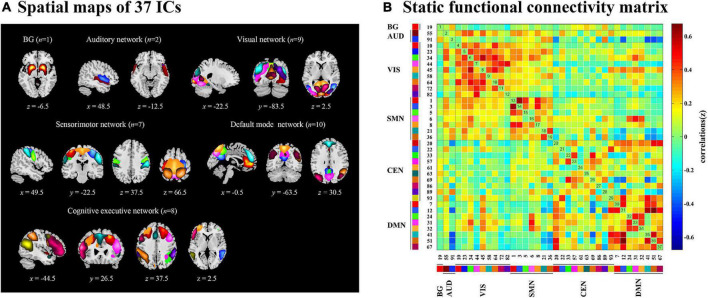
The 37 independent components identified by group independent component analysis. **(A)** Independent components spatial maps sorted into six functional networks. **(B)** Group-averaged static functional connectivity between independent component pairs was computed using the entire resting-state data. The value in the correlation matrix represents the Fisher’s z-transformed Pearson correlation coefficient. Each of the 37 independent components was rearranged by network group based on the six functional networks. BG, basal ganglia; AUD, auditory network; VIS, visual network; SMN, sensorimotor network; CEN, cognitive executive network; DMN, default mode network.

Out of the 100 independent components, 37 ICs were identified as meaningful according to the following criteria by Allen et al. (2014): (1) peak coordinates of spatial maps located primarily in the gray matter; (2) low spatial overlap with known vascular, motion, and susceptibility artifacts; (3) time courses dominated by low-frequency signals (ratio of the integral of spectral power < 0.10 Hz to.15–0.25 Hz); and (4) time courses characterized by a high dynamic range (a range difference between the minimum and maximum power frequencies). These 37 ICs were then sorted into six functional networks based on the spatial correlation values between ICs and the template and visual inspection (Shirer et al., 2012). The six functional networks were: basal ganglia (BG), auditory (AUD), visual (VIS), sensorimotor (SMN), cognitive executive (CEN), and default mode (DMN) (Figure 1A and Supplementary Table 1).

The following postprocessing steps were performed on the time courses of 37 ICs to remove remaining noise sources, including detrending linear, quadratic, and cubic trends, regressing out six realignment parameters and their temporal derivatives, despiking detected outliers by 3DDESPIKE, and low-pass filtering with a high cutoff frequency of.15 Hz (Allen et al., 2014). To obtain the static functional connectivity matrix, pair-wise Pearson’s correlations between ICs were computed and converted to z-scores *via* Fisher’s z-transformation using the post-processed time courses (Kim et al., 2017; Figure 1B).

### Dynamic Functional Connectivity

**FIGURE 2:**
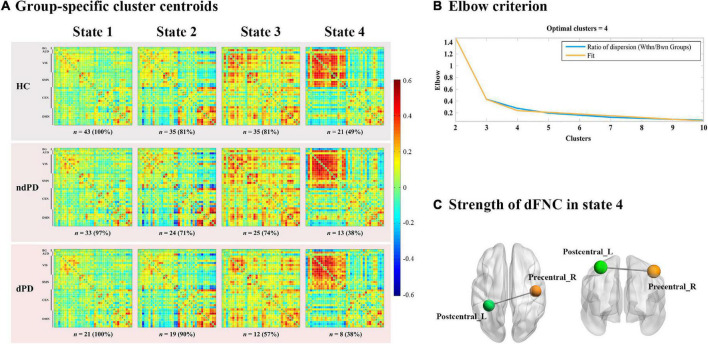
Functional connectivity state results. **(A)** Group-specific cluster centroids matrices for each state. Each subject can enter one to four of the defined states throughout the entire scanning. The number and percentage of subjects for each group entering the specific state were presented below the matrices. HC, healthy controls; ndPD, Parkinson’s disease without depression; dPD, Parkinson’s disease with depression. **(B)** Elbow criterion. The cluster validity index was computed as the ratio of the within-cluster distance to between-cluster distance. K = 4 combined the lowest cluster validity index and most well-balanced solution. **(C)** Strength of dynamic functional network connectivity (dFNC) in state 4. There were significant differences in state 4 among HCs, ndPD, and dPD groups (ANOVA, *P* < 0.001, FDR corrected) in strength of dFNC, located between left postcentral cortex (Postcentral_L) and right precentral cortex (Precentral_R).

Dynamic functional connectivity analysis was conducted using the GIFT toolbox through two steps: a sliding window approach and k-means clustering. First, as in previous studies (Kim et al., 2017; Fiorenzato et al., 2019; Chen et al., 2021), we applied a sliding time window of 22 TR (44 s) to segment the resting-state data, a setting that was proved to provide a good trade-off between the ability to resolve the dynamics of FC and the quality of the correlation matrix estimation (Allen et al., 2014), with a Gaussian window alpha value of 3 and a step of 1 TR, resulting in the analysis of 208 windows. To assess the robustness of the results regarding different window sizes, we repeated all the analyses for window sizes ranging from 20 to 28 TR (Supplementary Figure 3). For each window, a 37 × 37 pairwise covariance matrix was calculated by the regularized inverse covariance matrix (Varoquaux et al., 2010). Further, a penalty on the L1 norm of the precision matrix was imposed in the graphical LASSO framework with 100 repetitions to promote sparsity (Friedman et al., 2008). The resulting functional connectivity matrices were stabilized *via* Fisher’s z transformation and then residualized with age, gender, education, and MMSE scores. We finally obtained 208 functional connectivity matrices for each subject representing the dynamic changes of functional connectivity during the entire scan time for further analysis. Second, we applied the k-means clustering method to the windowed covariance matrices of all subjects to assess the reoccurring functional connectivity patterns (states), which were repeated 100 times (Allen et al., 2014). L1 (Manhattan) distance function was implemented considering its adaptation to high-dimensional data (Aggarwal et al., 2001). Based on the elbow criterion of the cluster validity index suggested by previous studies (Allen et al., 2014; Díez-Cirarda et al., 2018), the optimal number of clusters (k) was determined to be four (k = 4) (Figure 2B), and all matrices for each subject were then categorized into one of the four specific states. Thus, each subject can enter one to four of the defined states throughout the entire scanning (Figure 2A). Additionally, to assess the robustness of the results regarding the optimal value for k, we repeated the above analyses for k ranging from 2 to 5 (Supplementary Figure 4).

### Group Differences in Dynamic Connectivity: Temporal Properties and Strength

In order to explore the temporal properties of dynamic functional connectivity states, we calculated three different indices for each subject, including: (1) fraction time, defined as the percentage of total time one subject spent in a given state; (2) mean dwell time, defined as the time a subject remained in a certain state; and (3) number of transitions, defined as the number of translations between states. The differences among groups (HCs, ndPD, and dPD) were investigated using a one-way ANOVA test, followed by *post hoc* two-sample *t*-tests. *P*-values were corrected by false discovery rate (FDR) for multiple comparisons. The threshold for statistical significance was set at *P* < 0.05.

Additionally, the subject-specific connectivity pattern of each state was represented by the median value of all functional connectivity matrices assigned to that state. Group differences among patients with HCs, ndPD, and dPD in dynamic functional connectivity pairs (666 pairings) within each state were calculated using one-way ANOVA, followed by *post hoc* two-sample *t*-tests (*P* < 0.05, FDR-corrected).

### Dynamic Graph Theory Parameter Analysis

We applied a graph theory approach to analyze the dynamics of graph characteristics of the network using GRETNA software^4^. Thirty-seven ICs were defined as nodes and the functional connectivity between each two ICs was constructed as edges for each time window. The resulting 208 functional connectivity matrices per subject were binarized with respect to a fixed sparsity threshold (the existing number of edges divided by the maximum possible number of edges), set as 0.1 to 0.34 in 0.01 increments based on prior studies (Kim et al., 2017; Navalpotro-Gomez et al., 2020). Only positive correlations were considered in our analysis. We calculated the following parameters by integrating over all the defined threshold ranges within each time window, including small-worldness, global efficiency, betweenness centrality, clustering coefficient, and local efficiency of the network. Then the variance of these parameters over the time windows was computed to evaluate the dynamic graph properties for each subject. Two-sample *t*-tests (or Mann–Whitney U test) and ANOVA test (or Kruskal–Wallis test) were respectively conducted to assess whether there were differences in topological variability between PD and HCs groups or among the HCs, ndPD, and dPD groups (*P* < 0.05).

### Statistical Analyses

Statistical analyses were calculated using SPSS Statistic 24.0 (Chicago, IL, United States). A two-sample *t*-test or Mann–Whitney U test was applied to compare two groups. One-way ANOVA or Kruskal–Wallis test was performed to compare the HCs, ndPD, and dPD groups. A chi-squared test was used to compare categorical variables such as gender. Spearman’s correlation analyses were conducted to assess the correlations between the detected temporal properties and HAMD-17 scores. *P*-values were corrected for multiple comparisons and *P* < 0.05 was set as a threshold for statistical significance.

## Results

### Demographic and Clinical Characteristics

A total of 34 patients with ndPD, 21 patients with dPD, and 43 HCs were eventually included in the further analysis. There were no significant differences among the three groups in terms of age, gender, education, and MMSE scores (*P* = 0.963, *P* = 0.780, *P* = 0.150, *P* = 0.056). No significant differences between patients with dPD and ndPD were found in disease duration, H&Y stage, UPDRS-III scores (*P* = 0.823, *P* = 0.199, *P* = 0.097). In addition, the HAMD-17 score of the patients with dPD was significantly higher than that of patients with ndPD or HC (*P* < 0.001) (Table 1).

**TABLE 1:** Demographic and clinical information of the participants.

	dPD	ndPD	HCs	*P*-value
	*n* = 21	*n* = 34	*n* = 43	
Age (years)	59.33 ± 5.73	59.79 ± 8.40	59.44 ± 5.86	0.963^a^
Gender (male/female)	9/12	14/20	15/28	0.780^b^
Education (years)	9.05 ± 2.94	9.56 ± 2.67	10.51 ± 3.30	0.150^a^
Disease duration (years)	2.05 ± 1.40	1.97 ± 1.13	–	0.823^c^
H & Y	2.00(1.50, 2.00)	1.50(1.50, 2.00)	–	0.199^d^
UPDRS-III	28.57 ± 8.97	24.56 ± 8.28	–	0.097^c^
MMSE	27(25.5, 29)	29(28, 29)	29(27, 30)	0.056^e^
HAMD-17	17.43 ± 4.87	3.68 ± 1.75	1.21 ± 1.64	0.000^a^, *

*dPD, Parkinson’s disease with depression; ndPD, Parkinson’s disease without depression; HCs, healthy controls; H & Y stage, Hoehn and Yahr stages; UPDRS-III, the motor section of the Unified Parkinson’s Disease Rating Scale; MMSE, Mini-Mental State Examination; HAMD-17, 17-item Hamilton Depression Rating Scale. Parametric variables are presented as mean ± SD, and non-parametric variables are presented as median (interquartile range).*

*^a^One-way ANOVA.*

*^b^Chi-squared test.*

*^c^Two-sample t-test.*

*^d^Mann–Whitney U test.*

*^e^Kruskal–Wallis test.*

*P < 0.05.

### Intrinsic Connectivity Networks

Spatial maps of all 37 independent components defined by using group ICA are illustrated in Figure 1A. These 37 ICs made up the following six networks: BG (IC 19), AUD (IC 55, 91), VIS (IC 10, 23, 34, 44, 45, 58, 64, 72, 82), SMN (IC 1, 3, 5, 6, 8, 21, 36), CEN (IC 20, 22, 33, 57, 61, 63, 69, 86, 89, 93), and DMN (IC 7,12, 24, 31, 32, 41, 51, 67). The specific distribution of the 37 ICs is showed in Supplementary Table 1.

### Clustering Analysis and Dynamic Functional Connectivity State Analysis

**FIGURE 3:**
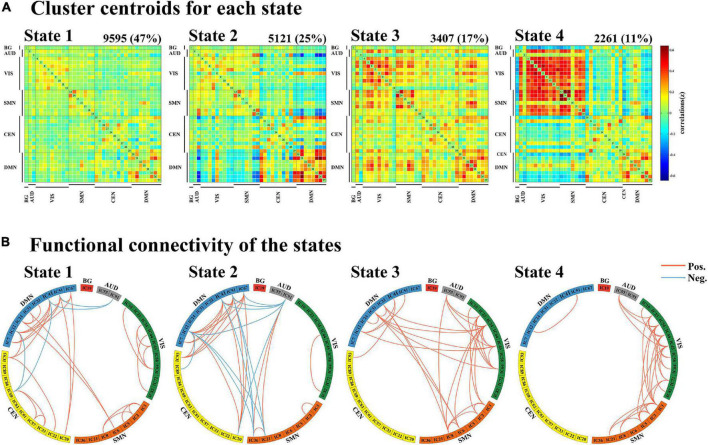
Results of the clustering analysis per state. **(A)** Resulting cluster centroids for each state. The total number of occurrences and percentage of total occurrences are listed above each cluster median. **(B)** Graphical representation of the strongest 5% functional network connections in each state. BG, basal ganglia; AUD, auditory network; VIS, visual network; SMN, sensorimotor network; CEN, cognitive executive network; DMN, default mode network.

Through the k-means clustering analysis, a total of four highly structured functional connectivity states were identified by the elbow criterion. We displayed the 5% of the functional connectivity network with the strongest positive or negative connections to observe the different patterns of each state (Figure 3B). Combined with the visualized cluster centroids (Figure 3A), state 1, which was the most frequent one (47%), was characterized by sparse connections both within and between networks, with relatively weak connectivity mainly located within DMN or VIS. State 2 (25%) was characterized by DMN-dominated and CEN-disconnected patterns, in which strong correlations were mainly exhibited within the DMN and between DMN and other networks (CEN/SMN/AUD), while the CEN showed sparse intra-network connections and inter-network connections with SMN/VIS/AUD. State 3 (17%) was characterized by relatively stronger positive connections within and between each state. State 4 (11%) was characterized by a regionally densely connected pattern, which showed positive correlations located mainly within VIS/SMN and between these two networks.

**FIGURE 4:**
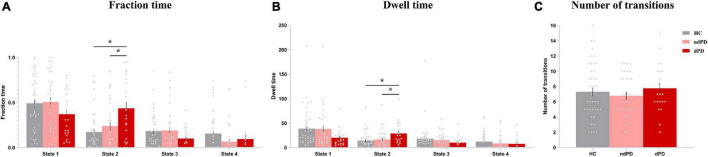
Differences in the temporal properties of dFC states among the three groups. **(A)** Fraction time. **(B)** Mean dwell time. **(C)** The number of transitions between states. Asterisks indicate a significant group difference (*P* < 0.05, FDR-corrected). Error bars represent the standard error. HC, healthy controls; ndPD, Parkinson’s disease without depression; dPD, Parkinson’s disease with depression.

One-way ANOVA indicated that there were significant differences between groups in terms of fraction time and mean dwell time in state 2 (ANOVA, fraction time: *P* = 0.004; mean dwell time: *P* = 0.048, FDR-corrected). *Post hoc t*-tests demonstrated that patients with dPD had a significantly higher fraction time in state 2 than that of patients with ndPD (*P* = 0.030 FDR-corrected) and healthy controls (*P* = 0.009, FDR-corrected). Additionally, patients with dPD spent significantly more dwell time in state 2. This is consistent with fraction time when compared to patients with ndPD (*P* = 0.048, FDR-corrected) and HC (*P* = 0.015, FDR-corrected). We found that fraction time (*P* = 0.006, r = 0.277) and mean dwell time (*P* = 0.035, r = 0.215) in state 2 were slightly correlated with HAMD scores in all participants when regressing out a group as a covariate. No differences were found in the number of state transitions or temporal properties in other states between the three groups (ANOVA, *P* > 0.05) (Figure 4 and Supplementary Table 2).

Validation analyses showed that the main results of temporal properties in state 2 were consistent for different window sizes (Supplementary Figure 3). Besides, with regard to different numbers of clusters, the main results also persisted, but there was a redundant state in k = 5, and the clustered states were incomplete for k less than 4 (Supplementary Figure 4).

### Strength of Dynamic Functional Network Connectivity

We subsequently examined between-group differences in the strength of connections for each state. ANOVA results (*P* < 0.001, FDR-corrected) showed a significant overall difference between the HC, patients with ndPD, and patients with dPD. *Post hoc* t-tests indicated that patients with dPD and patients with ndPD both had significantly decreased connection within the SMN (IC1/IC3, left postcentral cortex, and right precentral cortex) in state 4 compared to healthy controls (*P* < 0.001, FDR-corrected). In contrast, no differences were found between the ndPD and dPD groups (*P* = 0.58) (Figure 2C).

### Dynamic Graph Theory Properties

**FIGURE 5:**
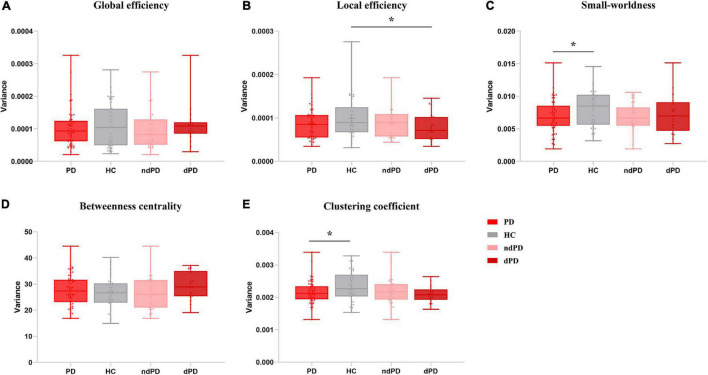
Variance of topological metrics in dynamic functional connectivity. **(A)** Global efficiency. **(B)** Local efficiency. **(C)** Small-worldness. **(D)** Betweenness centrality. **(E)** Clustering coefficient. Asterisks represent significant differences at *P* < 0.05. PD, Parkinson’s disease; HC, healthy controls; ndPD, Parkinson’s disease without depression; dPD, Parkinson’s disease with depression.

When comparing the variance of global efficiency, neither HCs group and PD group (Mann–Whitney U test, *P* = 0.755) nor HCs group and PD sub-groups (Kruskal-Wallis test, *P* = 0.552) differed significantly in this aspect. No significant differences were found between the HCs and PD groups (two-sample *t*-test, *P* = 0.501) or between the dPD, ndPD, and HCs groups (ANOVA, *P* = 0.286) with respect to betweenness centrality. In terms of the local efficiency of the network, there were significant differences between the dPD, ndPD, and HCs groups (ANOVA, *P* = 0.049). *Post hoc t*-tests revealed that the dPD group exhibited smaller variance than the HCs group did (*P* = 0.010), but no significant differences were found between the dPD and ndPD groups (*P* = 0.143) or between the ndPD and HCs groups (*P* = 0.319). There was a trend for less variability in the PD group than in the HCs group (two-sample *t*-test, *P* = 0.075). Further, the analysis of small-worldness variability demonstrated that the PD group was less variable than the HC group (two-sample *t*-test, *P* = 0.034), and there may be a trend of differences between the dPD, ndPD, and HC groups (ANOVA, *P* = 0.097), consistent with the results of clustering coefficient (two-sample *t*-test, *P* = 0.041; ANOVA, *P* = 0.076) (Figure 5 and Supplementary Table 2).

## Discussion

The present study investigated the characteristics of dynamic functional connectivity and dynamic topological properties in drug-naïve PD patients with depression, thereby excluding the confounding effects of dopaminergic therapy and long-term disease duration. Four distinct connectivity states were found across the entire participants. Importantly, we observed that in contrast to patients with ndPD and the healthy population, patients with dPD showed increased fractional time and mean dwell time in state 2, characterized by DMN-dominated and CEN-disconnected patterns. This indicated that patients with dPD preferred to spend more time in a dysfunctional connectivity state. Additionally, the above dynamic metrics exhibited a slight correlation with the severity of depression. Our results suggested that the occurrence of depression in PD may be attributed to the temporal fluctuations of functional network connectivity.

State 2 was characterized by strong connections mostly within the DMN or between DMN and several other networks. Accordingly, the DMN may play a central role in the development of depression in PD. The DMN is a highly integrated network that is active during wakeful rest state and deactivated during externally directed processes (Buckner et al., 2008), involved in self-referential processes and rumination (Belzung et al., 2015). Indeed, prior studies had proved that the DMN was implicated in numerous neurological and psychiatric disorders (Buckner et al., 2008; Leech and Sharp, 2014). Studies covering regional cerebral activity, functional connectivity, and large-scale network analysis have demonstrated that the DMN may facilitate the occurrence of depressive symptoms in PD (Luo et al., 2014; Wei et al., 2017; Liao et al., 2020). Sheline and colleagues found that failure to down-regulate activity within the DMN normally may result in dysregulation of automatic emotional processing, ultimately leading to depression (Sheline et al., 2009; Belzung et al., 2015). Furthermore, dynamic interactions between DMN and other networks influence cognition and emotion (Zhang and Volkow, 2019). In state 2, there were mainly strong positive couplings between DMN and CEN. The CEN is a task-positive network involved in multiple cognitive control functions including attention, memory, cognitive flexibility (Lerman et al., 2014; Dajani and Uddin, 2015), while it was proved to be anti-corrected with the DMN at rest in a healthy population (Fox et al., 2005). The disruption of the anti-correlation may be related to the abnormality of behavioral responses (Kelly et al., 2008). Besides, the relatively sparse connectivity within the CEN in our results was consistent with a former study which found that, in contrast to patients with ndPD, patients with dPD showed reduced functional connectivity in the core nodes of the CEN (Lou et al., 2015). The weak connections within the CEN or between CEN and SMN/VIS/AUD may impair the bottom-up and top-down modulation in emotion regulation (Phillips et al., 2003; Kaiser et al., 2015; Zhang and Volkow, 2019). Previous network dynamic analysis also found this top-down control was relevant to PD motor symptoms and could be improved after taking levodopa (Chen et al., 2021), which may further explain that the anti-parkinsonism medication also relieves depression in PD apart from the motor symptoms (Andersen et al., 2015). Our results suggested that the maintenance in DMN-dominated and CEN-disconnected state was linked to depression in PD patients, therefore, underlining the value of dynamic FC analysis in exploring the substrates of dPD.

Further, we observed that the ndPD group and dPD group relative to healthy controls showed weaker dynamic connectivity within the SMN in state 4, mainly located between the left postcentral cortex and right precentral cortex. PD was proposed as a disconnection syndrome (Göttlich et al., 2013). Decreased connection within the SMN was identified in patients with PD compared to HCs (Wang et al., 2021), and the results were validated in drug-naïve patients with PD in resting-state analysis (De Micco et al., 2021), supporting that the dysfunction of cortex-basal ganglia circuits participate in the PD pathophysiology (Lindenbach and Bishop, 2013; Burciu and Vaillancourt, 2018; Shen et al., 2020). Focused on the dynamics, our results provide further evidence of the significance of SMN abnormality in the mechanism of PD, in accord with a recent dFC study (De Micco et al., 2021). Nonetheless, no significant difference was observed between patients with dPD and ndPD. For one thing, dynamic functional connectivity abnormalities of SMN may just be a neuroimaging feature for PD, rather than for depression in PD. For another, it is possible that the disruption of functional integrity in SMN has not been aggravated enough to cause depression at the early stage of PD.

Global efficiency is a measure of parallel information transfer efficiency across the entire brain network (Achard and Bullmore, 2007), and dynamic global efficiency over time is considered as an efficient method to measure stability and flexibility of functional modulation of the whole brain in several neurodegenerative diseases (Schumacher et al., 2019). Kim and colleagues found higher variability of global efficiency in patients with PD (Kim et al., 2017), incompatible with our results. The local efficiency of the network is the average of the nodal local efficiency, reflecting the fault tolerance of the network when the index node is eliminated (Achard and Bullmore, 2007). Brain network tends to present economical small-worldness properties with high global and local efficiency (Latora and Marchiori, 2001). Betweenness centrality assesses the hubness of the whole brain despite the meaningless results in our study. The clustering coefficient of the network can quantify the local interconnectivity of the network and is also an important parameter measuring the small-worldness properties (Hou et al., 2020). In the present study, HCs showed significantly more variable local efficiency relative to the dPD group, along with higher variability of the small-worldness property and clustering coefficient than in the PD group, indicating that the rigidity and ineffectiveness of network communication in response to emotional demands appear to gradually expand from the local scale to the whole brain. In combination with our research and previous studies, the results of dynamic graph theory in PD are still controversial (Kim et al., 2017; Díez-Cirarda et al., 2018; Cai et al., 2019). The distinction in the disease stage could be a crucial cause, so further study covering different stages of PD will need to be conducted to explore the effects in PD physiology.

There still exists a few limitations in the present study that should be considered. First, only 21 patients with dPD were included in the current study. We did not observe a significant correlation between dynamic properties and HAMD scores when specifically considering the dPD group. This can be attributed to the small sample size, although we ruled out the effects of dopaminergic or antidepressant medication. Studies with larger sample sizes should be performed for further validation. Second, the participants in our study underwent single resting-state scanning, while a prior study suggested multiple sessions could improve the detection power especially under the circumstance of applying the sliding window approach (Hindriks et al., 2016). Third, although we had eliminated subjects with large head motion, matched mean framewise displacements between groups, and regressed out it as covariates, the effects of head motion in dFC analysis might not have been fully excluded.

## Conclusion

In summary, the present study is the first study to explore the characteristics of dynamic functional connectivity in *de novo* patients with PD and depression. Our study demonstrated that altered network connections over time, mainly involving the DMN and CEN, with less variable graph properties, could play important roles in the presence of depression in PD. Decreased time-varying FC within the SMN may facilitate the emergence of PD. Dynamic functional connectivity analysis could provide new insights into the pathophysiology of depression in patients with PD.

## Data Availability Statement

The original contributions presented in the study are included in the article/Supplementary Material, further inquiries can be directed to the corresponding authors.

## Ethics Statement

The studies involving human participants were reviewed and approved by the Medical Research Ethics Committee of the Affiliated Brain Hospital of Nanjing Medical University. The patients/participants provided their written informed consent to participate in this study.

## Author Contributions

WL designed and organized the research. JX, YL, LL, JR, and CP collected the imaging and assessment scale data. JX, MY, and HW were responsible for formal analysis. JX drafted the manuscript. WL and MY made important revisions to the manuscript. All authors contributed to the article and approved the submitted version.

## Conflict of Interest

The authors declare that the research was conducted in the absence of any commercial or financial relationships that could be construed as a potential conflict of interest.

## Publisher’s Note

All claims expressed in this article are solely those of the authors and do not necessarily represent those of their affiliated organizations, or those of the publisher, the editors and the reviewers. Any product that may be evaluated in this article, or claim that may be made by its manufacturer, is not guaranteed or endorsed by the publisher.

## Footnotes

1
http://rfmri.org/DPARSF

2
http://www.fil.ion.ucl.ac.uk/spm

3
http://icatb.sourceforge.net

4
http://www.nitrc.org/projects/gretna
