# Supplementary material for: Altered Dynamic Functional Connectivity in de novo Parkinson’s Disease Patients With Depression
Source: Front Aging Neurosci. 2022 Feb 14;13:789785. doi: 10.3389/fnagi.2021.789785 (PMC8882994; doi:10.3389/fnagi.2021.789785)
Supplement: Supplementary file 1 [file Data_Sheet_1.PDF]

Supplementary Material

Table S1 | Peak coordinates of 37 independent components

Intrinsic connectivity networks		Nv	T-value	MNI coordinate		
				x	y	z
**Basal ganglia network (BG)**						
IC19	R putamen	1135	24.9	21	9	-3
	L putamen	1096	26.8	-21	6	-6
**Auditory network (AUD)**						
IC55	R superior temporal gyrus	1465	24	60	-6	3
	L superior temporal gyrus	972	16.8	-60	-12	3
IC91	L superior temporal gyrus	1052	15.9	-57	6	-3
	R superior temporal gyrus	1741	22.8	57	3	3
**Visual network (VIS)**						
IC10	Bi middle occipital gyrus	1708	23.3	30	-93	0
IC23	R middle temporal gyrus	986	21.2	51	-60	3
	L middle temporal gyrus	842	18	-51	-69	6
IC34	Bi cuneus	1873	27.8	3	-81	36
IC44	L superior occipital gyrus	2491	20.0	-12	-90	24
	R superior occipital gyrus	242	8.1	21	-84	21
IC45	Bi lingual gyrus	2426	25	9	-51	0
IC58	R middle occipital gyrus	1023	18.3	33	-78	30
	L middle occipital gyrus	1198	22.9	-36	-84	24
	R fusiform gyrus	284	12.5	30	-45	-15
	L fusiform gyrus	317	13.5	-30	-42	-15
IC64	L cuneus	1600	24.9	0	-90	21
IC72	Bi calcarine	2990	22.4	3	-78	6
IC82	R superior occipital gyrus	2453	21	24	-87	21
	L superior occipital gyrus	249	9.1	-15	-96	15
**Sensorimotor network (SMN)**						
IC1	L postcentral gyrus	1936	23.1	-33	-39	57
IC3	R precentral gyrus	1455	24.5	45	-15	51
IC5	R precentral gyrus	1203	26.4	48	-9	30
	L postcentral gyrus	1133	24.0	-60	-6	18
IC6	Bi precuneus	839	21.9	0	-54	63
IC8	Bi paracentral lobule	1666	32.5	0	-18	63
IC21	R postcentral gyrus	1428	26.0	54	-24	45
	L Inferior parietal gyrus	806	15.7	-54	-30	45
IC36	Bi postcentral gyrus	2145	26.6	21	-48	63
**Cognitive executive network (CEN)**						
IC20	Bi middle frontal gyrus	4147	25.3	24	18	51
IC22	R middle temporal gyrus	1434	30.8	60	-48	9

	L middle temporal gyrus	359	14.0	-60	-51	6
	L middle frontal gyrus	225	6.2	-39	48	15
IC33	R Inferior parietal gyrus	1091	30.2	51	-42	45
	R middle frontal gyrus	533	11.1	42	45	6
	L Inferior parietal gyrus	326	10.8	-51	-45	48
IC57	R Inferior frontal gyrus	2046	27.5	42	15	27
	L Inferior frontal gyrus	1044	15.9	-48	15	30
	R Inferior parietal gyrus	345	13.1	39	-54	45
IC61	L middle frontal gyrus	3170	22.8	-30	54	9
	L precuneus	610	10.0	-6	-60	63
	R middle frontal gyrus	383	9.1	36	45	9
IC63	Bi supplementary motor area	2097	30.2	0	15	54
	Inferior frontal gyrus	396	11.8	-51	15	-3
IC69	L Inferior parietal	3500	24.7	-48	-42	42
IC86	L Inferior frontal gyrus	3159	22.1	-54	24	18
IC89	R supramarginal gyrus	936	29.8	57	-45	30
	L supramarginal gyrus	515	18.2	-54	-51	30
	Inferior frontal gyrus	379	10.3	51	15	-3
IC93	L middle temporal gyrus	3201	25.1	-54	-60	15
	R middle temporal gyrus	482	13.9	51	-54	18
**Default mode network (DMN)**						
IC7	Bi anterior cingulate	1628	29.1	-3	45	3
IC12	Bi precuneus	1369	33.4	-3	-57	24
IC24	Bi precuneus	1751	32.4	0	-57	45
IC31	Bi precuneus	1426	30.1	6	-72	42
IC32	Bi precuneus	1011	32.3	-3	-63	57
IC41	Bi superior medial frontal gyrus	2100	30.0	0	51	18
IC51	R angular gyrus	946	30.8	51	-57	30
	R middle frontal gyrus	857	11.7	39	21	39
	L angular gyrus	465	13.8	-45	-66	27
IC67	L Inferior parietal gyrus	1789	31.3	-36	-72	39
	R angular gyrus	498	20.7	39	-72	36

The coordinates are peak voxel coordinates of the one-sample t-test results for each independent component spatial maps of all subjects. $P<0.05$, FDR-corrected. Abbreviations: IC, Independent Component; Nv, number of voxel in each cluster; R, right; L, left; Bi, bilateral.

Table S2 | Results of temporal properties and dynamic graph theory parameters

		dPD	ndPD	HCs	P, uncorrected	P, FDR-corrected
Fraction time (%)	State 1	36.97±26.39	50.66±30.38	49.16±29.82	0.206[a]	0.206
	State 2	43.50±34.00	23.78±26.80	17.21±21.59	0.001[a]	0.004*
	State 3	10.00±11.75	18.95±22.94	18.22±19.05	0.201[a]	0.206
	State 4	9.52±20.91	6.60±13.83	15.41±22.01	0.133[a]	0.206
Mean dwell time	State 1	20.18±17.16	38.06±40.30	38.95±37.84	0.114[a]	0.228
	State 2	28.99±23.89	16.20±18.74	14.26±15.87	0.012[a]	0.048*
	State 3	10.14±12.24	15.25±13.91	18.36±27.97	0.345[a]	0.367
	State 4	7.63±12.79	8.25±14.44	12.33±16.34	0.367[a]	0.367
Number of transitions		7.76±3.21	6.79±2.93	7.30±3.77	0.577[a]	-
Variance of global efficiency ($\times 10^{-5}$)		10.85(8.51, 11.98)	8.23(5.12, 12.87)	10.40(4.91, 16.15)	0.552[b]	-
Variance of local efficiency ($\times 10^{-5}$)		7.12(5.13, 10.20)	9.02(5.73, 10.85)	8.93(6.70, 12.44)	0.049[b, *]	-
Variance of small-world ($\times 10^{-3}$)		6.96(4.69, 9.07)	6.64(5.48, 8.28)	8.49(5.64, 10.22)	0.097[b]	-
Variance of betweenness centrality		29.23±5.23	26.79±6.37	26.88±6.22	0.286[a]	
Variance of clustering coefficient ($\times 10^{-3}$)		2.10±0.25	2.19±0.40	2.33±0.46	0.076[a]	

Abbreviations: dPD: Parkinson's disease with depression; ndPD: Parkinson's disease without depression; HCs: healthy controls.

Parametric variables are presented as mean ± SD, and non-parametric variables are presented as median (interquartile range).

*P<0.05, FDR-corrected.

[a]One-way ANOVA.

[b]Kruskal-Wallis test.

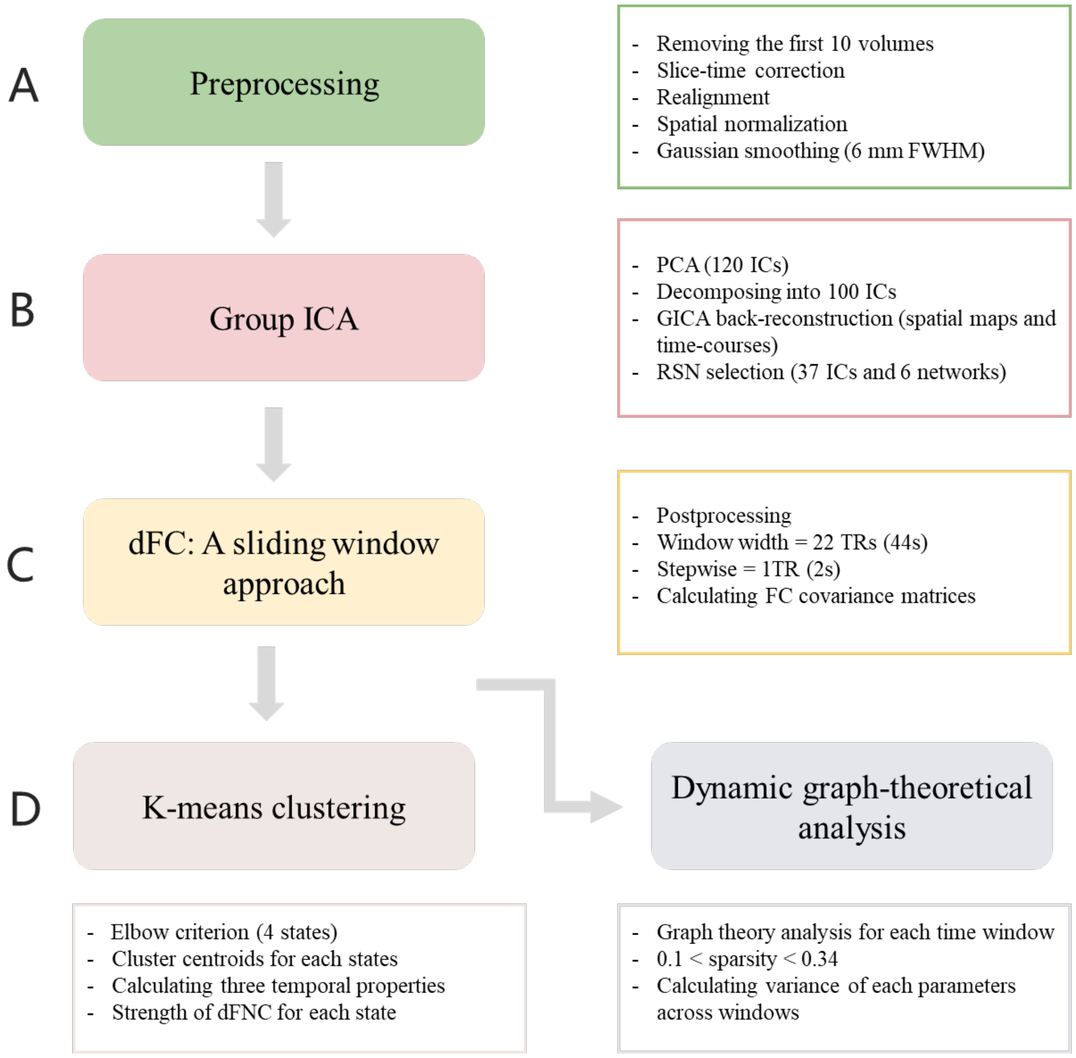

Figure S1 | Schematic of the analysis pipeline. (A) Preprocessing of resting state functional MRI data. (B) Group independent component analysis (ICA). (C) Computation for dynamic functional connectivity: A sliding window approach. (D) K-means clustering and dynamic graph-theoretical analysis.

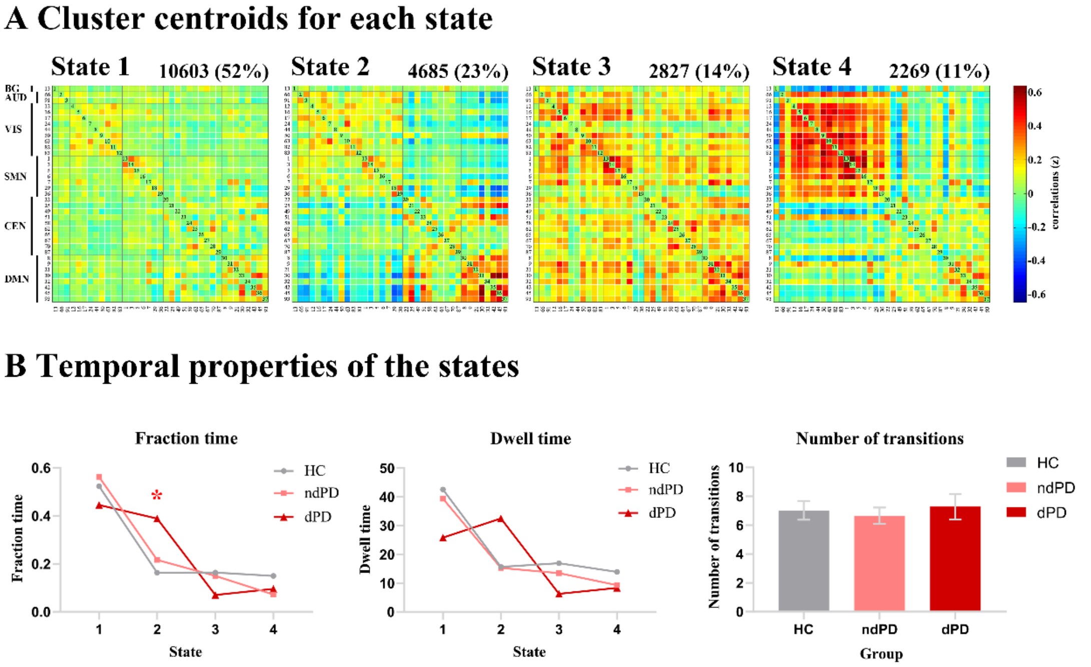

Figure S2 | Results for analyses after adding a scrubbing step in the preprocessing procedures. (A) Resulting cluster centroids for each state. The total number of occurrences and percentage of total occurrences are listed above each cluster median. (B) Differences in the temporal properties of dFC states among the three groups. Asterisks indicate a significant group difference ($P<0.05$, FDR corrected).

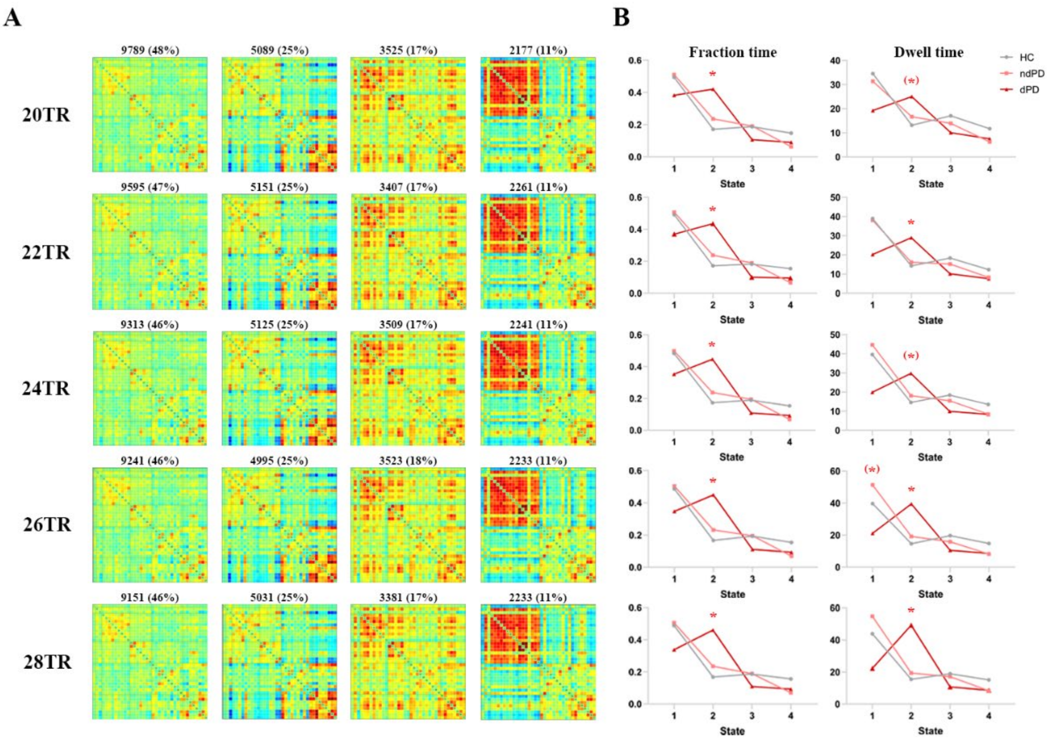

Figure S3 | Results for different window sizes. (A) Cluster centroids for each state in

different windows sizes. (B) the results of dynamic properties for different window sizes. Asterisks indicate a significant group difference (P<0.05, FDR corrected), while asterisks in parentheses indicate a significant group difference (P<0.05, FDR uncorrected).

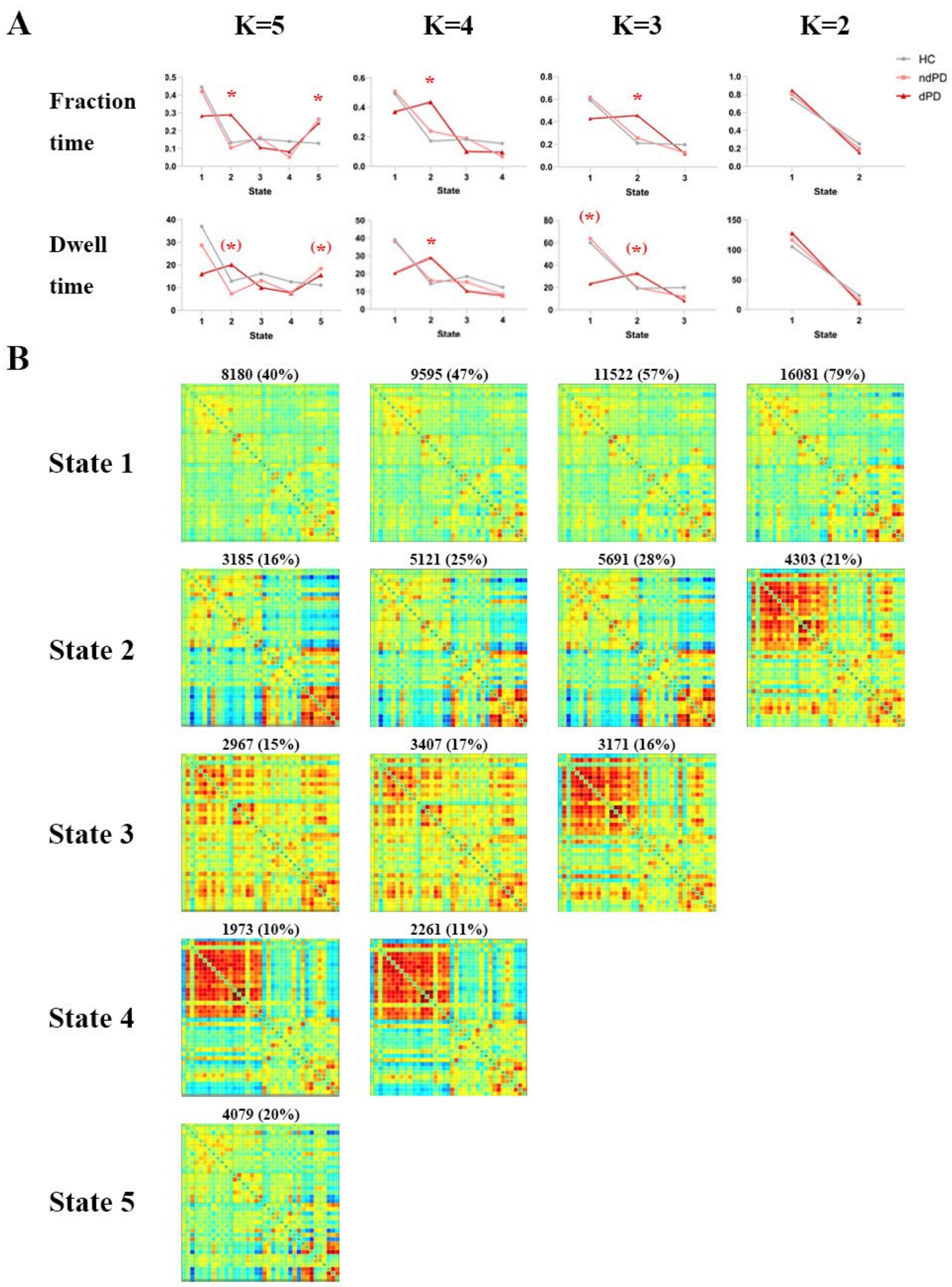

Figure S4 | Results for different numbers of clusters (K). (A) the results of dynamic properties for different numbers of clusters (K). Asterisks indicate a significant group difference (P<0.05, FDR corrected), while asterisks in parentheses indicate a significant

group difference (P<0.05, FDR uncorrected) (B) Cluster centroids for each state in different values of K.
